# Therapeutic TNF Inhibitors can Differentially Stabilize Trimeric TNF by Inhibiting Monomer Exchange

**DOI:** 10.1038/srep32747

**Published:** 2016-09-08

**Authors:** Karin A. van Schie, Pleuni Ooijevaar-de Heer, Lisanne Dijk, Simone Kruithof, Gertjan Wolbink, Theo Rispens

**Affiliations:** 1 Sanquin Research, Department of Immunopathology and Landsteiner Laboratory, Academic Medical Centre, University of Amsterdam, The Netherlands; 2 Jan van Breemen Research Institute | Reade, Amsterdam, The Netherlands

## Abstract

Tumor necrosis factor (TNF) is a homotrimeric cytokine that is a key mediator of inflammation. It is unstable at physiological concentrations and slowly converts into an inactive form. Here, we investigated the mechanism of this process by using a Förster resonance energy transfer (FRET) assay that allowed monitoring of monomeric subunit exchange in time. We observed continuous exchange of monomeric subunits even at concentrations of TNF high enough to maintain its bioactivity. The kinetics of this process closely corresponds with the appearance of monomeric subunits and disappearance of trimeric TNF in time at ng/ml concentrations as monitored by high-performance size-exclusion chromatography (HP-SEC). Furthermore, of the five therapeutic TNF inhibitors that are currently used in the clinic, three (adalimumab, infliximab, etanercept) were found to completely inhibit the monomer exchange reaction and stabilize TNF trimers, whereas golimumab and certolizumab could not prevent monomer exchange, but did slow down the exchange process. These differences were not correlated with the affinities of the TNF inhibitors, measured with both surface plasmon resonance (SPR) and in fluid phase using fluorescence-assisted HP-SEC. The stabilizing effect of these TNF inhibitors might result in prolonged residual TNF bioactivity under conditions of incomplete blocking, as observed *in vitro* for adalimumab.

Tumor necrosis factor (TNF) is a homo-trimeric cytokine that plays a key role in mediating inflammation^1^. It is produced as a transmembrane molecule from which the soluble TNF is released via proteolytic cleavage. Noncovalent interactions hold the monomeric units together in a compact, bell-shaped trimer^2^,^3^. Both soluble and membrane-bound TNF can signal via two different receptors, TNF receptors I and II. For both receptors, signaling proceeds via a complex of three receptor molecules binding to the trimeric TNF^4^,^5^.

Interestingly, it has been reported that (soluble) TNF is non-stable at physiological concentrations (pg/mL-ng/mL) and slowly converts into inactive, presumably monomeric TNF, both in buffer and serum^6^,^7^. This process is apparently (partially) reversible^7^,^8^, in line with studies that demonstrate that denatured TNF can re-fold into an active, trimeric protein^8^. The spontaneous conversion into an inactive form might play a role in constraining the signaling of this very potent cytokine after being released in the active, trimeric form. The details of this process have not been fully elucidated, amongst others, due to the paucity of assays that can monitor homo-multimerization reactions.

TNF is also an important mediator of a number of inflammatory auto-immune disorders including rheumatoid arthritis, Crohn’s disease, and psoriasis^1^. In many patients, the inhibition of TNF activity via a blocking antibody or antibody-receptor fusion protein has proven to successfully suppress disease activity, and may even lead to clinical remission^9^,^10^,^11^. There are currently five TNF inhibitors approved for use in the clinic: three full-length antibodies (infliximab, adalimumab, and golimumab), a PEGylated Fab fragment (certolizumab pegol), and a receptor-Fc construct (etanercept), see Fig. 1^12^,^13^. All TNF inhibitors block the binding of TNF to its receptors, which explains the mechanism of action. However, little is known about the fate of TNF-anti-TNF complexes, their stability, size, rate of clearance, and uptake by antigen-presenting cells, which might contribute to the immunogenic potential of the TNF inhibitors.

Here, we studied the mechanism of dissociation of TNF into monomeric subunits. We adapted a Förster resonance energy transfer (FRET) assay that was previously used to monitor antibody subunit exchange in human IgG4 molecules (Fab arm exchange)^14^. Using this assay, we demonstrate that continuous monomer exchange takes place even at high concentrations of TNF. Furthermore, we used this assay to study the effects of the different TNF inhibitors on the stability of TNF trimers.

## Results

### TNF monomer exchange

**Figure 2:**
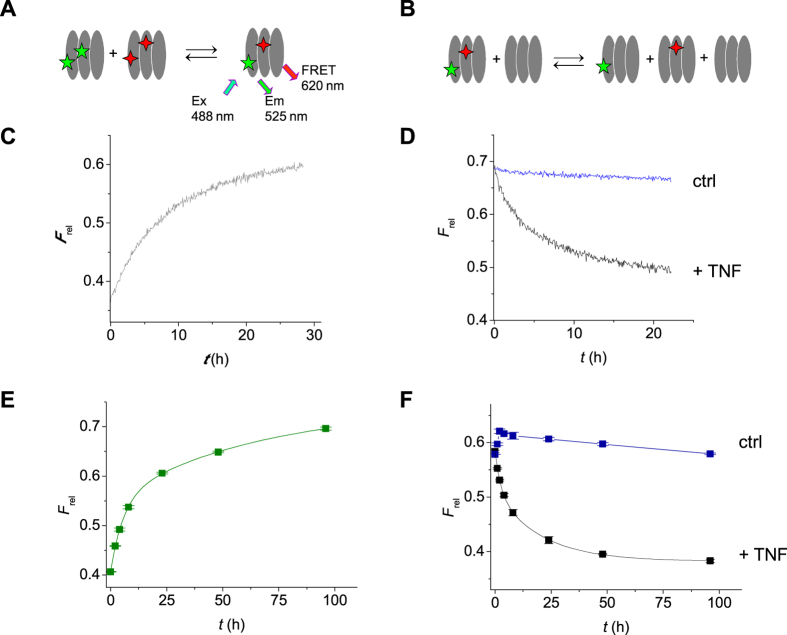
Kinetics of monomer exchange of TNF. (**A,C,E**) TNF (0.5 μg/mL), fluorescently labeled with DyLight-488 (TNF-488) or DyLight-594 (TNF-594), both with a degree of labeling of ca. 2, will exchange monomeric units resulting in trimeric TNF molecules that contain both 488- and 594-labeled monomers (**A**). These molecules will generate a FRET signal at 620 nm following excitation at 488 nm, which can be monitored in real-time (**C**). The process was also monitored for extended times by consecutive measurements (**E**). (**B,D,F**) TNF trimers that acquired monomeric subunits with both types of fluorescent label as described in A will generate a FRET signal (**B**). If an excess of unlabeled TNF is added, the fluorescently labeled monomers will ‘dilute’ over many TNF trimers resulting in a loss of FRET signal in time (**D,F**). This also represents the process of monomer exchange. Blue line represents control (ctrl) where no unlabeled TNF is added. (**C,D**) representative examples of triplicate experiments, (**E,F**) average of duplicate experiments; error bars indicate SEM. *F*_rel_ = *F*_620_/*F*_588_. Ex = excitation, Em = emission.

In order to investigate the putative dissociation and re-association of TNF subunits, we made use of a FRET assay. TNF was fluorescently labeled with either DyLight-488 or DyLight-594 such that on average ca. 2 dye molecules were attached to TNF (Fig. 2A). Both species were mixed and incubated at 37 °C at ca. 1 μg/mL, a concentration at which TNF is reported to be ‘stable’. A FRET signal developed in time, demonstrating the formation of TNF species that incorporate both types of dye, indicative of the exchange of monomers (Fig. 2C). Monitoring the reaction for extended times revealed bi-phasic kinetics (*t*_1/2_ = ca. 3–5 and 20–50 hours; Fig. 2E). The reaction kinetics was not significantly affected by choosing a two-fold lower or higher concentration (not shown). In order to exclude the alternative possibility that aggregation (as well as artificial associations due to the fluorescent labeling) were causing the appearance of a FRET signal, an excess of unlabeled TNF was introduced after 4 days of incubation of TNF-488 and TNF-594 (Fig. 2B). As expected, the FRET signal slowly disappeared again, with similar kinetics, indicative of fluorescently labeled TNF monomers exchanging with unlabeled TNF thereby ‘diluting’ the labeled fraction over the unlabeled molecules (Fig. 2D,F). Thus, trimeric TNF appears to continuously dissociate into monomeric (and possibly dimeric) units that can re-associate.

### Stability of TNF trimers

**Figure 3:**
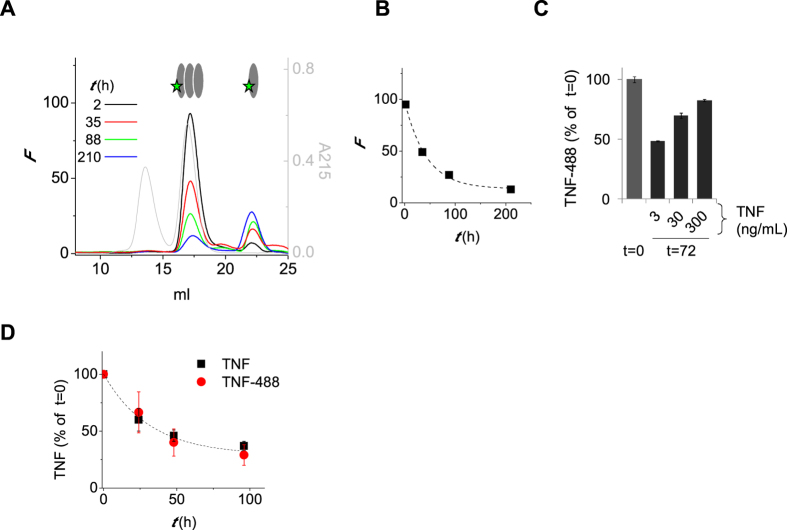
Stability of trimeric TNF. (**A**) TNF-488 (DOL 0.6; 3 ng/mL) was incubated at 37 °C and samples were analyzed by HP-SEC. Column was calibrated using a sample of IgG and Fab fragments (resp. 150 and 50 kDa; grey line, detected at 215 nm). Monomeric TNF was only partially recovered. (**B**) Disappearance of trimeric TNF-488 (3 ng/mL) in time. (**C**) TNF-488 (3 ng/mL) was incubated alone or in the presence of 27 or 297 ng/mL of unlabeled TNF for 72 hrs and recovery of trimeric TNF-488 was evaluated as in (**A**). (**D**) TNF or TNF-488 (3 ng/mL) was incubated at 37 °C for various times and analyzed using an ELISA that only measures trimeric TNF. (**A–C**) representative of n = 2; D average of triplicate experiment, error bars indicate SEM. *F* = fluorescence.

Next, we investigated the dissociation of TNF trimers. TNF-488 was incubated at 3 ng/mL for various times at 37 °C and analyzed using high-performance size-exclusion chromatography (HP-SEC), monitoring the elution of TNF by in-line fluorescence detection. Disappearance of trimeric TNF in time was observed, accompanied by the appearance of monomeric TNF (Fig. 3A). However, recovery of total TNF was not complete, indicating that monomeric TNF may have precipitated, or otherwise, remained on the column. The time-course of dissociation roughly corresponded to that observed for the monomer exchange (Fig. 3B). In the presence of increasing amounts of (unlabeled) TNF, the dissociation was diminished (Fig. 3C). This is consistent with a dynamic process of monomer exchange, with faster re-association at higher TNF concentrations preventing progressive loss of trimeric and monomeric TNF by further unfolding. The disappearance of trimeric TNF furthermore corresponded to the disappearance of activity measured in a TNF ELISA (Fig. 3D).

### TNF inhibitors can stabilize TNF

**Figure 4:**
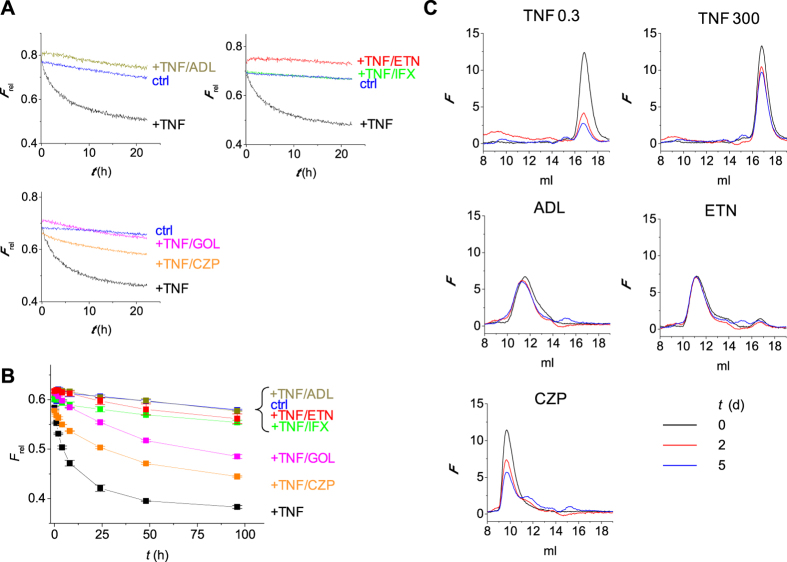
Influence of TNF inhibitors on TNF stability. (**A,B**) Mixed TNF-488/594 (0.5 μg/mL, incubated at 37 °C for 7 days) was incubated at 37 °C with 3 μg/mL unlabeled TNF as described in Fig. 2B, and monomer exchange was monitored as the disappearance of FRET signal either in real-time to cover the initial kinetics (**A**) or by consecutive measurements for the slower kinetics (**B**). Different TNF inhibitors were added at 100 μg/mL. Adalimumab (ADL), etanercept (ETN), and infliximab (IFX) blocked monomer exchange to undetectable levels, whereas golimumab (GOL), and certolizumab pegol (CZP) reduced the rate of monomer exchange. In the control experiment (ctrl), no TNF was added. Shown in (**A**) are representative examples, in (**B**) the average of duplicate experiments. (**C**) TNF-488 (0.3 ng/mL) was incubated for up to 5 days at 37 °C and analyzed as described in Fig. 3A (n = 2). Addition of 300 ng/mL unlabeled TNF largely prevented loss of trimer. The addition of 1 μg/mL of adalimumab or etanercept to 0.3 ng/ml TNF-488 resulted in the formation of TNF-containing complexes that remained stable during the 5-day incubation. However, complexes formed with 1 μg/mL of certolizumab pegol slowly degraded. *F*_rel_ = *F*_620_/*F*_588_. *F* = fluorescence.

Next, we investigated the influence of therapeutic TNF inhibitors on the stability of TNF. Monomer exchange was evaluated by monitoring the ‘dilution’ of mixed and equilibrated TNF488/TNF594 after addition of unlabeled TNF in the presence or absence of an excess of TNF inhibitor. This approach was chosen because binding of several TNF inhibitors to TNF was influenced by the fluorescent labeling (Figure S1). Under the conditions of these experiments, i.e., an excess of TNF inhibitor over TNF, the dominant type of complex will be a 3:1 complex of inhibitor:TNF (except for ETN). Adalimumab, infliximab, and etanercept were found to completely abrogate the monomer exchange (Fig. 4A,B). On the other hand, certolizumab and golimumab resulted in a somewhat slower monomer exchange but did not prevent it. In other words, whereas the former TNF inhibitors stabilize the TNF trimer, the latter do not or only slightly so. To confirm these results, stability of complexes formed between TNF-488 and adalimumab, certolizumab, or etanercept was monitored using HP-SEC (Fig. 4C). Whereas complexes of TNF with adalimumab or etanercept were completely stable for up to 5 days at 37 °C, those formed with certolizumab were not. Similar results were obtained with adalimumab Fab (Figure S2).

### Affinities

**Figure 5:**
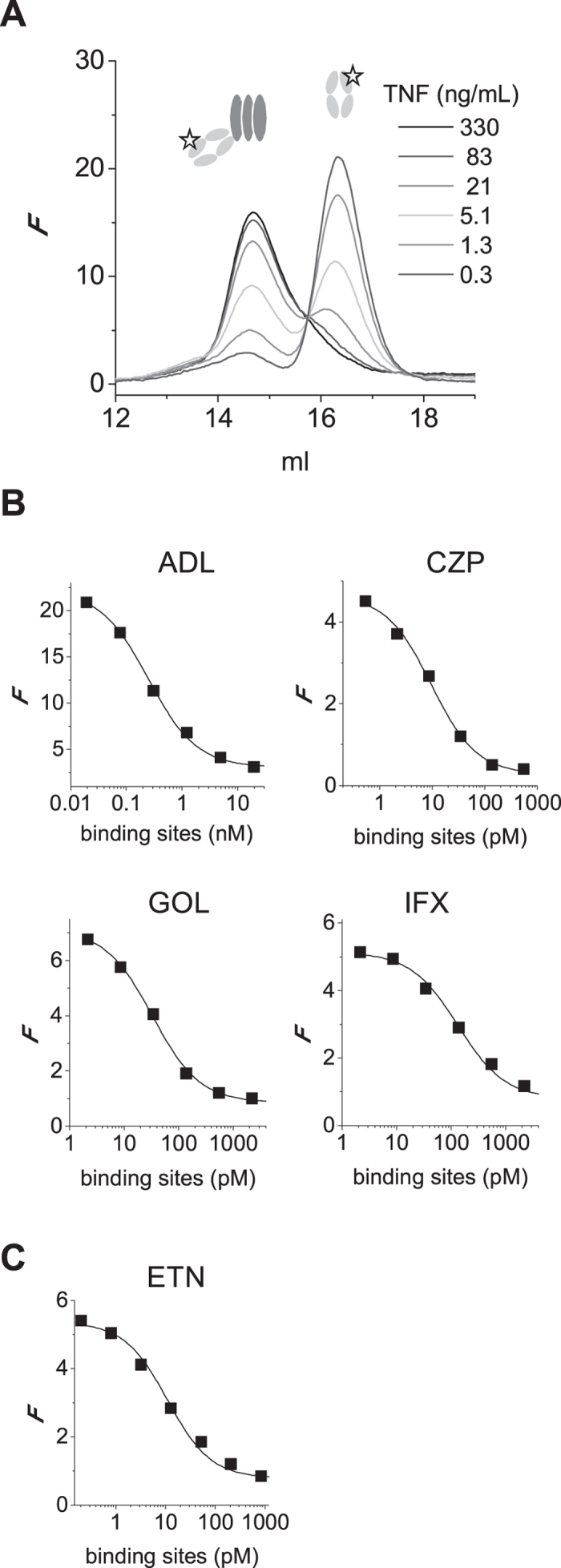
Affinities of TNF inhibitors in fluid phase. (**A**) Example of fluid-phase affinity measurement of TNF-anti-TNF. DyLight-488 labeled adalimumab Fab’ is incubated with different concentrations of TNF and analyzed by HP-SEC, monitoring the elution of bound and free adalimumab Fab’-488 by in-line fluorescence detection. (**B**) Free Fab-488 is plotted vs the concentration of TNF (molar concentration of binding sites) and a 1:1 Langmuir binding model is fitted, thus assuming independent association of anti-TNF Fab’s to each binding site on TNF. (**C**) TNF-488 was incubated with different concentrations of etanercept and analyzed analogously to B, using the molar concentration of etanercept in the calculations. Representative of n = 3. *F* = fluorescence.

The differential stabilizing activity of the different TNF inhibitors may be caused by differences in affinity. To investigate this possibility, affinities were measured by two methods. First, binding of TNF to TNF blockers captured onto a biosensor chip was monitored by surface plasmon resonance (SPR). This method has previously been used by others to measure the affinities of several of the TNF blockers, but not all five of them together, and with sometimes widely different results between studies^15^,^16^,^17^,^18^,^19^,^20^,^21^. One possible complication with this set-up is the fact that both the antibodies and the TNF are multivalent, and depending on the exact experimental design, the measurements might not be fully representative of intrinsic affinities. Therefore, we also determined affinities in fluid phase by monitoring binding between TNF and fluorescently labeled Fab fragments of the TNF blockers using fluorescence-assisted HP-SEC as described previously (refs 22 and 23; Fig. 5). For etanercept, binding is assumed to be 1:1 and a reverse titration experiment was carried out. The results are summarized in Table 1. Overall, both methods are in good agreement, with the exception of certolizumab, for which the fluid phase affinity is substantially higher than results from SPR (Table 1). The stabilizing effects of the TNF blockers do not correlate with the differences in affinity; for instance, adalimumab has the lowest affinity but can stabilize TNF trimers, whereas certolizumab has one of the highest affinities but does not stabilize the trimer.

### Adalimumab Fab’ can preserve TNF bioactivity

**Figure 6:**
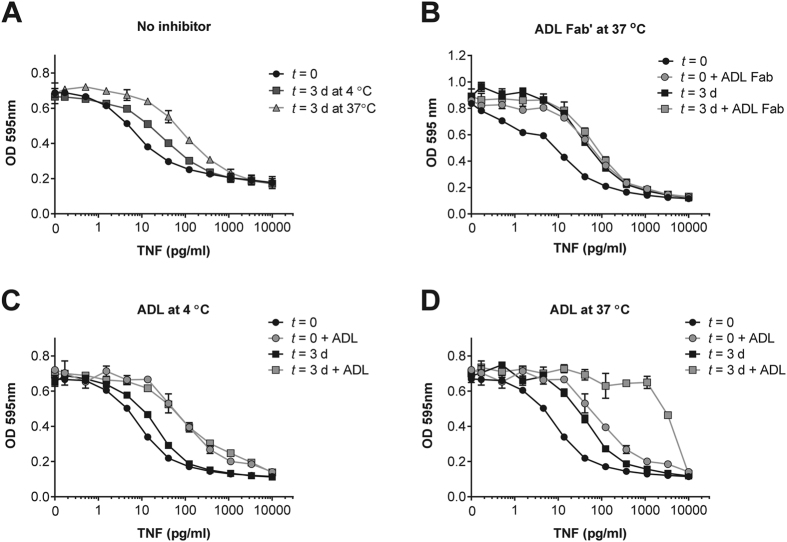
Influence of time, temperature and the TNF inhibitor adalimumab on TNF bioactivity. (**A**) Bioactivity of a titration of TNF was measured immediately or after three days of incubation at 4 °C or 37 °C using a TNF sensitive WEHI bioassay. (**B**) Effect of adalimumab Fab’ on TNF activity, determined immediately or after three days of incubation at 37 °C. (**C,D**) Effect of adalimumab on TNF activity, measured immediately or after three days of incubation at 4 °C (**C**) or 37 °C (**D**). All graphs show representative data of two (**A–C**) or four (**D**) experiments.

By suppressing TNF monomer exchange, TNF inhibitors stabilize TNF in its trimeric form, possibly leading to prolonged TNF bioactivity, analogously to soluble TNF receptor^24^. To investigate this, we measured TNF bioactivity using WEHI cells. First, we measured TNF bioactivity after three days of incubation at 4 °C or 37 °C in absence of TNF inhibitors. As expected, a considerable amount of TNF bioactivity was lost after three days of incubation at 37 °C and to a smaller degree when TNF was incubated at 4 °C (Fig. 6A). Next, the stabilizing effect of adalimumab Fab’ on TNF was examined. TNF was incubated in the presence of adalimumab Fab’ at a concentration that was anticipated to result in partial saturation of TNF based on the *K*_d_. Bioactivity was analyzed immediately or after three days incubation at 37 °C. As expected, adalimumab Fab’ reduced TNF bioactivity due to its competition with cell bound TNF receptors. However, after incubation for three days at 37 °C, additional loss of TNF bioactivity was almost completely prevented (Fig. 6B). These results suggest that through stabilization of the trimeric form of TNF, adalimumab Fab’ can partially preserve TNF bioactivity. Adalimumab was found to similarly stabilize TNF bioactivity at 4 °C (Fig. 6C), but after incubation at 37 °C, activity dropped to very low values (Fig. 6D).

## Discussion

The present study elucidates the dynamic nature of native, trimeric TNF molecules, which continuously dissociate into monomers, which may or may not reassociate depending on the overall concentration. This links the previously observed loss of activity upon incubation at low concentrations to a mechanistic framework that describes this process in terms of monomer dissociation and reassociation. In line with studies of homo-dimerization reactions of IgG4^14^,^22^, the rate of the TNF dissociation process is essentially independent of the concentration of TNF. The rate of reassociation on the other hand depends strongly on the overall concentration and is the decisive factor in maintaining bioactivity at high concentrations but not at low concentrations.

Interestingly, we revealed pronounced differences in the mode of binding of the five therapeutic TNF inhibitors currently in use in the clinic, with several markedly stabilizing the TNF trimer. Although these therapeutic TNF inhibitors block the binding of TNF to the receptors upon binding, we also observed a contradicting prolonged residual TNF bioactivity upon incubation with adalimumab Fab’, most likely due to this stabilizing effect. These results resemble a study that demonstrates an augmented TNF activity in the presence of soluble TNF receptor, which also stabilizes TNF at low concentrations^24^. Clinically, this could mean that in case the concentrations of TNF inhibitor drop to the point that TNF is incompletely blocked, for instance in case a patient develops anti-adalimumab antibodies^25^, TNF activity could emerge that might persist longer than if no TNF inhibitor would have been present. In contrast to adalimumab Fab’, adalimumab appeared to greatly diminish TNF bioactivity after a three day incubation at 37 °C. Similar incubation of TNF with adalimumab at 4 °C demonstrated a stabilizing activity as seen with adalimumab Fab’. These results are puzzling and may point to an artefact in the assay used to assess bioactivity of TNF. Alternatively, immune complex precipitation might be induced by cross-linking of TNF by adalimumab. Adalimumab and TNF are described to have the tendency to form complexes containing three adalimumab and three TNF trimers when incubated at 37 °C at near-equimolar ratio’s, suggesting that these complexes are thermally most stable^15^. Formation of such complexes would significantly alter the competition between adalimumab and TNF receptors, and thus influence the bioactivity of TNF.

**Figure 7:**
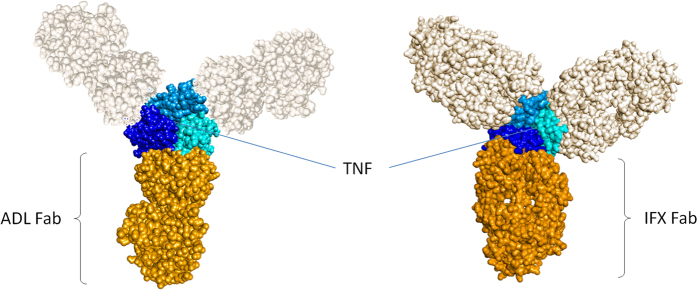
Models of TNF in complex with (**A**) adalimumab Fab and (**B**) infliximab Fab (top view). Adalimumab binds an epitope comprising two TNF monomers. Infliximab binds an epitope comprising a single TNF monomer. Based on PDB structures 3WD5 and 4G3Y^17^,^26^.

The inhibition of monomer exchange of the TNF inhibitors adalimumab, infliximab, and etanercept is most likely due to their respective modes of binding. For adalimumab, a crystal structure of the Fab in complex with TNF demonstrates that the epitope that is recognized comprises two subunits of TNF^26^. Because of the three-fold symmetry of TNF, this epitope is present three times. In other words, all TNF subunits are held together upon binding of adalimumab (Fig. 7A). A previous study demonstrated a stabilizing effect of adalimumab on TNF towards denaturation with urea^3^, which is in line with our present findings. For infliximab, a crystal structure of its complex with TNF reveals only direct contacts between the Fab and one TNF monomer (Fig. 7B). However, infliximab stabilizes a loop that is flexible in unbound TNF, thereby possibly stabilizing interactions between the TNF monomers^17^. For golimumab and certolizumab, binding to TNF resulted in a slower exchange of TNF monomers, but the process was not abrogated. Both TNF inhibitors bind with high affinity, excluding weaker binding as an explanation for the lack of stabilization. Instead, both molecules probably bind to an epitope that comprises only a single TNF monomer. Indeed, disappearance of (trimeric) certolizumab-TNF complexes was accompanied by the appearance of a smaller type of complexes (Fig. 4C), tentatively a TNF monomer subunit in complex with 1 molecule of certolizumab. In contrast to infliximab, certolizumab and golimumab might bind to a TNF monomer without significantly influencing its conformation and thereby the interactions between the TNF monomers.

Interestingly, in competition experiments, replacement of radiolabeled TNF bound to etanercept by an excess of unlabeled TNF was previously found to be a relatively fast process^27^. This might be due to the fact that whereas the overall binding strength of etanercept to TNF is determined by its two receptor arms, each individual arm may bind weakly, characterized by fast dissociation, facilitating replacement of bound TNF in conditions of excess of free TNF. In the present study, we observe that despite this apparent possibility of fast (partial) release of TNF by etanercept, trimeric TNF is stabilized by etanercept up to the point that no monomer exchange was observed over the course of four days.

The bi-phasic kinetics of the monomer exchange reaction suggests heterogeneity in the TNF structure. We obtained similar results using recombinant TNF from two different suppliers, but cannot exclude the possibility that this heterogeneity is a non-physiological feature of these particular TNF products. This biphasic kinetics is not apparent from the HP-SEC analysis nor from previous studies^7^, which can be attributed to the superior precision of the FRET assay. The fluorescent labeling itself might introduce artefactual changes to the TNF molecule. However, the fact that similar kinetics was observed for monitoring exchange between two types of labeled TNF, as well as exchange of labeled TNF with unlabeled TNF (Fig. 2A,B) suggests that the labeling does not significantly affect the exchange process. The labelling is carried out at high concentrations of TNF, such that the trimeric conformation predominates and the association surfaces between the monomeric units are actually shielded. Therefore, it is unlikely that labels will attach to sites that substantially affect the multimerization. In fact, labeling of a protein complex can be a general trick to avoid incorporation of labels that can affect the interactions between the constituents of such a complex (including antibody-antigen complexes).

The stabilizing activity of TNF inhibitors such as adalimumab or infliximab might have practical implications. Accurate measurements of cytokines in biological samples is challenging^28^, and TNF has been a notoriously difficult cytokine to accurately quantify in fluids such as serum. In fact, in multiple studies, correlation between different assays - including commercially available ones - was often poor and sometimes lacking altogether^29^,^30^,^31^. As a result, while many studies have reported TNF levels across diseases and populations, it is impossible to compare results from one study with another, or decide which of the reported TNF concentrations are truly representative of a given condition. One of the factors that may contribute to the variable results is TNF dissociation during sampling, storage, and freeze-thawing of samples, in combination with a variable sensitivity of immunoassays for trimeric and monomeric TNF. By adding a stabilizing TNF antibody to freshly isolated serum samples, much of these issues might in theory be circumvented. Of course, this would require an assay that is not influenced by the presence of the TNF inhibitor.

In summary, using a FRET assay we demonstrated continuous exchange of TNF monomers between native TNF homotrimers. The process can be blocked by the therapeutic TNF inhibitors adalimumab, infliximab, and etanercept, but not by certolizumab and golimumab.

## Materials and Methods

### Materials

Recombinant human TNF was obtained from Life Technologies and Active Bioscience. Both were supplied as lyophilized powder and reconstituted as recommended by the supplier. TNF concentrations were determined by TNF ELISA (Sanquin, The Netherlands). Recombinant therapeutic TNF blockers used in this study are adalimumab (Humira, Abbott), infliximab (Remicade, MSD), golimumab (Simponi, MSD), certolizumab pegol (Cimzia, UCB Pharma), and etanercept (Enbrel, Pfizer). The first three TNF blockers are full-length IgG1 kappa antibodies, certolizumab pegol is a Fab’ fragment conjugated to a polyethylene glycol (PEG) group, and etanercept is a receptor-Fc construct.

Full-length IgG1 certolizumab was made by cloning constructs coding for VH and VL of certolizumab^21^ and the constant domains of human kappa and human IgG1 genes (obtained from GeneArt, Invitrogen) into a pcDNA3.1 (Invitrogen) essentially as described before^22^. Expression vectors were used for transient transfection of HEK293F cells with 293fectin and OptiMEM (Invitrogen), using the Freestyle HEK293F expression system (Invitrogen) according to the instructions supplied by the manufacturer. Cell culture supernatants were centrifuged for 15 minutes at 1700 G, followed by loading on a Protein G column (Protein G 4 fast flow, GE Healthcare) and elution of the certolizumab IgG1 with 0.1 M glycine, pH 2.5. The eluate was neutralized immediately with 2 M Tris-HCl, pH 9 and dialyzed overnight to PBS (10 mM sodium phosphate pH 7.4; 140 mM sodium chloride). After dialysis, samples were sterile-filtered and stored at −20 °C. Specificity of the full-length certolizumab was confirmed with a certolizumab-specific ELISA (Sanquin Biologicals).

### Generation of F(ab’)2, Fab’, and Fab fragments

F(ab’)2 and Fab’ fragments were generated using pepsin, and subsequent reduction and alkylation with dithioerythritol and N-ethylmaleimide respectively, as described before^32^. Fab fragments were generated using papain digestion as described previously^33^. In case of certolizumab pegol, papain digestion removes the C-terminal part of the Fab’ fragment including the site of PEG attachment. Certolizumab Fab was purified by gel filtration.

### Fluorescent labeling

TNF, Fab and Fab’ fragments were fluorescently labeled with DyLight 488 or DyLight 594 amine reactive dye (Pierce/Thermo Scientific). Unreacted dye was removed by repeated dilution/concentration using Amicon Centriprep centrifugal filter devices (Millipore, Billerica, MA, US) until no dye could be detected anymore in the filtrate. The average degree of labelling (DOL) ranged from 0.3 - 3.

### Kinetic measurements (FRET)

Kinetics of the exchange reaction of TNF monomers were monitored in real-time using a previously developed FRET assay for the monitoring of non-covalent protein subunit exchange^14^. Briefly, the reactions were carried out using one of the following protocols: A) 0.5 μg/mL of TNF-488 and 0,5 μg/ml TNF-594 (final TNF concentration 1 μg/ml) in PBS-I (PBS containing 1 mg/mL IVIG (Nanogam, Sanquin, The Netherlands)) were incubated at 37 °C, and the reaction was initiated by mixing equal volumes of both solutions into a thermostatted quartz cuvette (37 °C). B) A solution of 0.25 μg/ml of TNF-488 and 0.25 μg/ml TNF-594 (final TNF concentration 0.5 μg/ml) in PBS-I was incubated in the dark for 7 days at 37 °C. Then the reaction was initiated by adding several microlitres of a concentrated stock solution of (unlabeled) TNF to a final concentration of 2 μg/mL. TNF blockers were similarly added from concentrated stocks as appropriate to a final concentration of 100 μg/mL. Kinetics was monitored at 37 °C by measuring the appearance of a FRET signal using a Varian Cary Eclipse fluorescence spectrophotometer equipped with a thermostatted multi-cell holder (excitation 488 nm, emission 620 nm; emission at 588 nm was used to account for baseline drifts, 588 nm being the isosbestic point in the overlay spectrum; relative fluorescence (*F*_rel_) is determined by dividing emission at 620 nm by emission at 588 nm).

### Fluorescence-assisted high-performance size-exclusion chromatography (HP-SEC)

Stability of TNF was assessed by incubating TNF-488 at 0.3–3 ng/mL in PBS-I for various times between 0 and 5 days, alone or in the presence of unlabeled TNF (30 or 300 ng/mL), adalimumab, certolizumab pegol, or etanercept (1 μg/mL) in the dark at 37 °C. Samples were subsequently analyzed applying 500 μl to a Superdex 200 HR 10/300 column (GE Healthcare, Uppsala Sweden) at 0.5 ml/min using a thermostatted autosampler (4 °C), which was connected to an ÄKTAexplorer HPLC system (GE Healthcare, Uppsala Sweden). Elution profiles of TNF-488 were monitored by measuring the fluorescence (excitation/emission 488/525 nm) with a Prominence RF-20Axs on-line fluorescence detector (Shimadzu, Kyoto, Japan).

### Affinity measurements using fluorescence-assisted HP-SEC

Affinities were determined as described previously^23^. Briefly, serial four-fold dilutions of TNF (0.01–500 ng/ml) were incubated with a fixed concentration of 50–200 pg/ml of Fab-488 (certolizumab, golimumab) or Fab’-488 (adalimumab, infliximab) in PBS-I. Samples (1000 μl) were applied using a thermostatted autosampler (25 °C) to a Superdex 200 HR 10/300 column at 0.5 ml/min (GE Healthcare, Uppsala Sweden), which was connected to an ÄKTAexplorer HPLC system (GE Healthcare, Uppsala Sweden). Elution profiles of Fab-488 were monitored by measuring the fluorescence (excitation/emission 488/525 nm) with a Prominence RF-20Axs on-line fluorescence detector (Shimadzu, Kyoto, Japan). To calculate dissociation constants, fluorescence at 17 ml, corresponding to the peak maxima of free Fab-488, respectively, were plotted against the concentration of TNF (molar concentration of the number of binding sites) and a 1:1 binding model was fitted to the data using Microcal Origin software. Alternatively, TNF-488 (DOL of 0.6) was incubated with serial fourfold dilutions of etanercept and analyzed likewise.

### Surface plasmon resonance measurements

Surface Plasmon Resonance measurements were performed using a Biacore T-200 instrument (Biacore AB, Breda, The Netherlands) at 25 °C. Mouse anti-human IgG (MH16-1, Sanquin, The Netherlands) was immobilized at a concentration of 10 μg/ml in 10 mM sodium acetate, pH 5.0, on a CM5 sensor chip using *N*-hydrosuccinimide/1-ethyl-3-(3dimethylaminopropyl)carbodiimide hydro-chloride (NHS/EDC) at a flow rate of 15 μL/min. Anti-TNF monoclonal antibodies were dissolved at 5 μg/ml in PBS containing 0.05% polysorbate-20 (PBS-T), and passed through the cells at 30 μL/min yielding ca. 100 response units (RU) of bound antibody. Subsequently, binding of TNF (50–300 ng/mL) dissolved in PBS-T was measured at a flow rate of 30 μL/min for 600 s followed by monitoring dissociation for another 600 s. After each run, immobilized ligand was regenerated by removing bound analyte with 5 μL of 0.1 M phosphoric acid. Adsorptions obtained in the reference channel without bound anti-TNF antibody was subtracted from the adsorptions in the other cells. Association and dissociation kinetics were fitted using the models provided with the Biacore analysis software.

### WEHI bioassay

TNF bioactivity was determined using a WEHI bioassay. Per well 40.000 WEHI-164 cells were plated in IMDM (BioWhittaker) containing 5% FCS (Bodinco), 100 U/ml penicillin, 100 μg/ml streptomycin (both from Gibco), 1 μg/ml Actinomycin D and 50 μM β-Mercapto-ethanol (both from Sigma). TNF (range 0–20 000 pg/ml TNF, Active Bioscience) was incubated in presence or absence of adalimumab Fab’ (13.3 or 26.6 ng/ml) or adalimumab (20 ng/ml) and either used immediately or incubated for three days at 4 °C or 37 °C. Subsequently, TNF, TNF-adalimumab Fab’ or TNF-adalimumab samples were 1:1 added to the WEHI-164 cells, resulting in 0–10 000 pg/ml TNF, 6.6 ng/ml or 13 ng/ml adalimumab Fab’ and 10 ng/ml adalimumab as a final concentration. WEHI-164 cells were incubated for 24 h at 37 °C and 5% CO_2_ after which cell viability was measured using the MTT-reduction method. MTT (Sigma, diluted in 0.14 M NaCl and 0.01 M HEPES) was added to the cell cultures in a final concentration of 0.83 mg/ml and incubated for four hours, after which SDS (Gibco, diluted in 0.01 M HCl) was added to a final concentration of 5% for overnight incubation. Adsorption was measured at 595 nm and as reference 670 nm using a plate reader (Synergy 2, BioTek).

## Additional Information

**How to cite this article**: van Schie, K. A. *et al.* Therapeutic TNF Inhibitors can Differentially Stabilize Trimeric TNF by Inhibiting Monomer Exchange. *Sci. Rep.* **6**, 32747; doi: 10.1038/srep32747 (2016).

## Supplementary Material

Supplementary Information

## Figures and Tables

**Figure 1:**
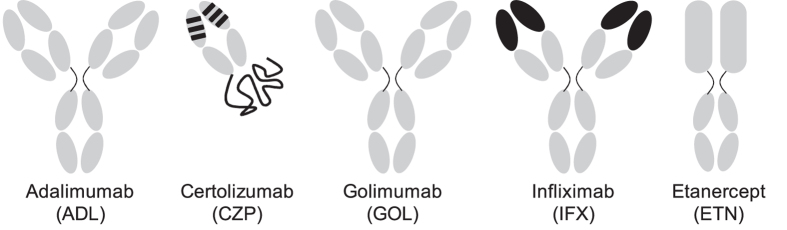
Structure of the different TNF inhibitors. Adalimumab (ADL) is a fully human IgG1 kappa antibody, as is golimumab (GOL). Certolizumab pegol (CZP) is a pegylated humanized Fab’ fragment, infliximab (IFX) a chimeric antibody, and etanercept (ETN) a receptor-Fc construct. Human origin is shown in grey, murine origin in black.

**Table 1:** Binding of TNF inhibitors to TNF at 25 °C.

TNF inhibitor	*k*_a_ (10^6^ M^−1^ s^−1^)^a^^,^^b^	*k*_d_ (10^−4^ s^−1^)^a^	*K*_d_ (pM)^a^^,^^b^	*K*_d_ (pM)^b^^,^^c^
adalimumab	0.72 (0.02)	1.3 (0.3)	183 (40)	244 (53)
certolizumab	3.2 (0.5)	1.5 (0.6)	71 (35)	9 (4)
golimumab	1.9 (0.2)	1.2 (0.3)	59 (20)	26 (6)
infliximab	1.7 (0.2)	2.4 (0.5)	138 (30)	123 (11)
etanercept	11 (2)	1.7 (0.7)	16 (8)	11 (3)^d^

^a^Measured using Biacore (n = 2–5).

^b^Association rates and dissociation constants are expressed relative to the molar concentrations of actual binding sites on TNF, which equals 3 for adalimumab, certolizumab, golimumab, and infliximab; for etanercept, 1:1 binding was assumed. To compare results to papers where molar trimeric TNF concentrations were used, multiply *k*_a_ and divide *K*_d_ by a factor of three for the mAbs.

^c^Measured in fluid phase by fluorescence-assisted HP-SEC (n = 3).

^d^Determined in reverse experiment in which etanercept was titrated to TNF-488.
