# Supplementary material for: Therapeutic TNF Inhibitors can Differentially Stabilize Trimeric TNF by Inhibiting Monomer Exchange
Source: Sci Rep. 2016 Sep 8;6:32747. doi: 10.1038/srep32747 (PMC5015024; doi:10.1038/srep32747)
Supplement: Supplementary Information [file srep32747-s1.doc]

# Therapeutic TNF Inhibitors can Differentially Stabilize Trimeric TNF by Inhibiting Monomer Exchange

Karin A. van Schie, Pleuni Ooijevaar-de Heer, Lisanne Dijk, Simone Kruithof, Gertjan Wolbink, Theo Rispens

**Supplemental Figure S1.**

Labeling of TNF with DyLight-488 influences the binding of the different TNF inhibitors to various degrees. A comparison is made of complexes formed upon mixing TNF-488 (average degree of labeling 0.6 or 1.5) with an excess of TNF inhibitor. Whereas etanercept and certolizumab appear to be not affected, binding of adalimumab is slightly affected and binding of golimumab and infliximab is more drastically affected.

**Supplemental Figure S2.**

TNF-488 (0.3 ng/mL) was incubated for 4 days at 37 ^o^C in the presence of of 1 μg/mL of adalimumab Fab and analyzed as described in Figure 3A.
